# Factors Influencing Job Burnout and Musculoskeletal Disorders among Coal Miners in the Xinjiang Uygur Autonomous Region

**DOI:** 10.1155/2021/6629807

**Published:** 2021-02-12

**Authors:** Huijun Deng, Dingsheng He, Fuye Li

**Affiliations:** ^1^College of Public Health, Xinjiang Medical University, Urumqi, China; ^2^Fuzhou Center for Disease Control and Prevention, Fuzhou, China

## Abstract

**Background:**

Work-related musculoskeletal diseases (WMSDs) have been associated with job burnout. Currently, few studies have investigated the relationship between job burnout and WMSDs among coal miners.

**Methods:**

In this cross-sectional study, 1,325 staff were selected from 6 coal mining companies using a stratified cluster sampling method. The Chinese version of “Musculoskeletal Questionnaire” and “Occupational Burnout Scale” were used to investigate the link between WMSDs and job burnout. Logistic regression was conducted to analyze the factors influencing WMSDs.

**Results:**

A total of 1,500 questionnaires were distributed, with a response rate of 88.33%. The prevalence of WMSDs in coal miners was 65.58%, while the prevalence of total, mild, moderate, and severe burnout were 90%, 39.77%, 43.77%, and 6.49%, respectively. The average score for job burnout was 50.78 ± 11.93. The prevalence of WMSDs among coal miners varied significantly with the length of service (*χ*^2^=14.493, *P*=0.001), type of work (*χ*^2^=11.438, *P*=0.022), shift system (*χ*^2^=6.462, *P*=0.040), and annual income (*χ*^2^=6.315, *P*=0.043). The proportions of male coal miners with moderate and severe burnout were 45.1% and 6.8%, respectively, which were higher compared with 28.6% and 2.9%, respectively, for women. The proportion of male coal miners with mild burnout was 38.1%, which was lower compared with 59.0% for women (*P* < 0.05). Coal miners who work more than two shifts had the highest burnout, while those who work day shifts had the lowest burnout (*P* < 0.001). The prevalence of WMSDs in the severe burnout group and in 9 body locations was significantly higher than that in other burnout groups (*P* < 0.001). Logistic regression results showed that length of service, type of work, annual income, and burnout level are associated with WMSDs among coal miners (*P* < 0.05).

**Conclusions:**

The prevalence of job burnout and WMSDs among coal miners in Xinjiang is relatively high. Job burnout is a risk factor for WMSDs among coal miners.

## 1. Introduction

Work-related musculoskeletal diseases (WMSDs) are injuries or diseases affecting muscles, bones, and nerves caused by exposure to risk factors (including bad social psychosocial factors) associated with occupational activities in the workplace [1]. The diseases mainly manifest as pain, discomfort, and obstruction of related activities. WMSDs are common occupational health problems in various industries [2, 3]. The treatment of these diseases not only incurs huge medical expenses [4, 5] but also affects daily work and life [6]. A study found that 28%–84% of waist and upper limb injuries are associated with psychosocial factors [7]. However, most people in China believe that WMSDs are a physical hazard occurring only in other countries and thus ignore the impact of nonphysical harm (social psychological hazards) on the risk of WMSDs [8]. Job burnout is a social psychological factor that was first proposed by Freudenberger in 1974 [9]. He defined it as a syndrome of emotional and physical exhaustion [10], divided into emotional exhaustion (physical and mental fatigue), depersonalization (negativity, indifference), and three dimensions of personal accomplishment (lack of work feeling) [11]. In recent years, work pressure and tension have increased dramatically in China, leading to a high prevalence of job burnout [12]. Several studies have found that job burnout not only makes the body tired but also leads to WMSDs [3, 13, 14]. Indeed, exhaustion dimension of burnout was positively associated with musculoskeletal pain [14]. Currently, coal mines in China account for about 13.9% of the total coal mines in the world. There are nearly 4 million people engaged in underground coal mine operations [15]. Xinjiang is one of the main coal mining regions in China. Coal miners are a special occupational group. They are exposed to challenging mining conditions for a long time, making them not only suffer from serious job burnout [16] but also a high-risk group likely to develop WMSDs (their waist has the most severe WMSDs [17] followed by shoulders and knees [4]). Burnout and WMSDs are likely to increase accident rates among miners [17], which decreases the productivity and safety of miners. Therefore, this study aimed to determine the correlation between job burnout and WMSDs among the miners. This will reveal the causes of WMSDs among coal miners and provide ideas for effective occupational health management and intervention for miners.

## 2. Materials and Methods

### 2.1. Participants

The study was approved by the Ethical Committee of the First Affiliated Hospital of Xinjiang Medical University. Study participants were enrolled from six coal mining enterprises that were selected based on the annual coal output. The annual coal output is divided into large, medium, and small, with random sampling being used to select two enterprises from each category in Xinjiang. A total of 1,325 coal miners were enrolled. The inclusion criteria were (1) informed consent and voluntary participation in this survey; (2) formal employment in a coal mine; (3) 18 years old ≤ age < 60 years old; length of service ≥1 year; (4) no physical examination, mental illness, and genetic history; and (5) no blood relationship between the subjects. Participants with nearly 20% incomplete data were excluded from the study. In total, 1,500 questionnaires were distributed, but 175 were eliminated because they did not meet the inclusion criteria. In the end, 1,325 valid questionnaires were obtained from respondents, representing a response rate of 88.33%.

### 2.2. Data Collection

The following data were collected using the questionnaire: (i) Self-designed general survey: the age, gender, ethnicity, education level, length of service, type of work, and marital status of coal miners are included. (ii) Job burnout questionnaire: The measurement of job burnout was based on the newly developed Yongxin Li's job burnout questionnaire by Fuye Li. The scale is highly reliable and valid with a retest reliability of 0.843 and Cronbach's *α* coefficient of 0.780 [18, 19]. The scale has 3 dimensions (emotional exhaustion, depersonalization, and personal accomplishment) and 15 questions (5 questions for each dimension). The questionnaire was assigned to Likert level 7. The range from 1 to 7 represented “never” to “every day,” and some (3, 6, 9, 12, 15 questions) were scored in reverse order. Based on 25 scores for emotional exhaustion, 11 scores for depersonalization, and 16 scores for personal achievement, job burnout levels were divided into zero burnout (all below 3 thresholds), mild burnout (only above 1 threshold), moderate burnout (only above 2 thresholds), and severe burnout (all above 3 thresholds). (iii) Questionnaire for WMSDs: The Chinese version of the musculoskeletal questionnaire jointly developed by Yang et al. [5, 20] and others was used to identify symptoms of any part of the body in the first 12 months, including the following soft parts of the body: back, shoulders, neck, elbows, lower back, hands/wrists, hips, knees, and ankles/feet.

### 2.3. Statistical Analysis

Data collected from the questionnaire were entered into the EpiData 3.1 database and checked for consistency. The data were analyzed using the 21.0 version of IBM SPSS statistical software. Quantitative data were described by mean ± standard deviation (‾*x* ± SD), and categorical variables were analyzed using the *χ*^2^ test. Simple and multivariable adjusted logistic regression models were used to assess the association between job burnout and musculoskeletal symptoms. Bilateral test level *α*=0.05. *P* < 0.05 was considered to be statistically significant.

## 3. Results

### 3.1. Subject Characteristics

A total of 1,325 coal mining enterprise workers (1325/1500, 88.33%; male: 1220 (92.1%) and female: 105 (7.9%)) completed the study questionnaire. The age of the workers ranged from 18 to 60 years with an average of 42.15 ± 8.65 years, while the length of service ranged from 1 to 42 years with an average of 17.48 ± 10.66 years. The average job burnout score of coal miners was 50.78 ± 11.93, and the rate of job burnout was about 90.0%. The average score for emotional exhaustion was 16.83 ± 7.77 points, while for depersonalization 12.61 ± 6.90 points, and for personal accomplishment 21.34 ± 7.66 points.

### 3.2. The Relationship between Different Demographic Characteristics and the Prevalence of WMSDs

The prevalence of WMSDs among coal miners varied significantly with length of service (*χ*^2^=14.493, *P*=0.001), type of work (*χ*^2^=11.438, *P*=0.022), shift system (*χ*^2^=6.462, *P*=0.040), and annual income (*χ*^2^=6.315, *P*=0.043). The prevalence of WMSDs among coal miners increased with the length of service. When the length of service was 10∼20 years, the OR value was 1.643 (*P* < 0.05). With regard to different shift systems, the prevalence of WMSDs among workers with more than two shifts was lowest with an OR value of 0.659 (*P* < 0.05). Transport workers had the highest prevalence of WMSDs among the different types of work, with an OR value of 1.373 (*P* < 0.05). The prevalence of WMSDs was highest in the group of workers earning 15,000∼70,000 yuan compared with the other income categories with an OR value of 1.521 (*P* < 0.05) (Table 1).

### 3.3. Comparison of Job Burnout Grades with Different Demographic Characteristics

The number of male coal miners with moderate and severe burnout was 550 (45.1%) and 83 (6.8%), respectively, which was higher than 30 (28.6%) and 3 (2.9%), respectively, for women. There was a statistically significant difference between the number of male and female coal miners who had mild burnout, 465 (38.1%) and 62 (59.0%) (*P*=0.001), respectively.

The degree of burnout among coal miners varied significantly with shift systems. The highest degree of burnout was observed in coal miners who had more than two shifts, while the lowest degree of burnout was observed in coal miners on day shifts (*P* < 0.001) (Table 2). Overall, 527 (39.77%) coal miners had mild burnout, 580 (43.77%) had moderate burnout, whereas 86 (6.49%) had severe burnout.

### 3.4. Correlation between Different Levels of Job Burnout and WMSDs

The prevalence of WMSDs in the severe burnout group and in 9 body locations (neck, shoulders, back, elbows, lower back, hands/wrists, hips, knees, and ankles/feet) was significantly higher than that in other burnout groups (*P* < 0.001). This shows that the prevalence of WMSDs differs among different levels of burnout, suggesting that the level of burnout is a risk factor for WMSDs among coal miners. The risk of WMSDs in miners with mild burnout is 2.306 times higher than that of miners with zero burnout (*P* < 0.05). For miners with moderate burnout, the risk of WMSDs is 2.459 times higher than that of miners with zero burnout (*P* < 0.01). For miners with severe burnout, the risk of WMSDs is 2.965 times higher than that of miners with zero burnout (*P* < 0.001). The prevalence of WMSDs in the miners was highest in the waist followed by neck, shoulders, and knees (Tables 3 and 4).

### 3.5. Multivariate Logistic Regression Analysis of Factors Influencing WMSDs

Coal miner demographic variables (age, length of service, shift system, type of work, annual income) and burnout levels (zero burnout, mild burnout, moderate burnout, severe burnout) with *P* < 0.1 in the single factor analysis were incorporated into the logistic regression equation. Demographic variables and the level of burnout were used as independent variables, while the prevalence of WMSDs (nondiseased group = 0, diseased group = 1) was used as a dependent variable to establish a WMSDs regression model. The length of service, type of work, annual income, and level of burnout were found to influence the risk of WMSDs among coal miners (*P* < 0.05). Further analysis showed that the 11–20 years length of service group was more likely to develop WMSDs compared with the 1–10 years length of service group. Analysis of the influence of annual income on WMSDs showed that the 70000–100000 yuan income group was more likely to develop WMSDs compared with the 15000–70000 yuan income group. Similarly, a higher burnout level was associated with a higher risk of WMSDs. The values of individual factors are shown in Table 5, and the logistic regression results are shown in Table 6.

## 4. Discussion

This study investigated the relationship between burnout in coal miners and the development of WMSDs for the first time. Previous reports from China indicate that the prevalence of WMSDs among miners is 61% [21]. In this study, we found that the prevalence of WMSDs among coal miners was 65.58% and that the prevalence increased with length of working years of the coal miners [22]. This is because working in coal mining industry for long duration increases the risk of WMSDs, which eventually affects the physical and mental health of miners [4, 17]. With regard to shift systems, the prevalence of WMSDs in miners working for two shifts exceeded that of miners working for more than two shifts, suggesting that working for two shifts increases the risk of WMSDs. This is because working hours for miners with more than two shifts may be shorter (usually 12 hours for two shifts and 8 hours for three shifts), which gives them enough time to rest and hence relieve muscle fatigue. In a single factor analysis, the OR of WMSDs for miners working for more than two shifts was the lowest. This is because working for more than two shifts (including three shifts and four shifts) require that one works for few hours, indicating that such miners have enough time to rest. However, in the binary logistic regression analysis, it is found that shifting does not affect the risk of WMSDs significantly.

Analysis of the influence of the different types of jobs on the prevalence of WMSDs showed that transportation workers had the highest prevalence of WMSDs, which is consistent with the results of a previous study [4]. WMSDs have been reported in coal miners who operate coal mine trucks and other mobile equipment. This is because coal transportation workers are expected to maintain a high degree of concentration, operate locomotives for long periods of time with limited range of motion, and engage in repetitive operations that are likely to tire the muscles. On the other hand, it has been observed that injuries may not be due to repetition and posture problems but may be as a result of the coal transporter hitting something in the cab due to vibrations and bumps caused by the unevenness of the ground of the coal mine [4]. Moreover, the prevalence of WMSDs was highest in the 15,000∼70,000 yuan income group. This may be due to the fact that the low-income groups are generally new or short-term miners who are unfamiliar with the working environment and are prone to accidents, and the burden of labor tasks is also relatively higher.

In this study, the rate of job burnout among coal miners was 90%. Mild burnout was 39.77%, moderate burnout was 43.77%, and severe burnout was 6.49%, which is similar to a report by Bauernhofer et al. [23]. A comparison of the levels of job burnout between male and female coal miners showed that moderate and severe burnout for males were 45.1% and 6.8%, respectively, which were higher than moderate burnout (28.6%) and severe burnout (2.9%) in females. This is because male miners engage in arduous tasks, greater psychological pressure, and fewer approaches to vent. Therefore, they are more prone to job burnout. Coal miners who worked more than two shifts had the highest level of burnout, while those who worked on day shifts had the lowest level of burnout. This is because too many shifts result in inadequate sleep, disordered biological clock, and lack of motivation [24].

We also found that the prevalence of WMSDs in the severe burnout group and its 9 sites was much higher than that in other burnout groups. The prevalence of WMSDs was highest in the waist, followed by shoulders, neck, and knees [17], because the waist is an important connection between the upper and lower limbs of the human body. During coal mining, coal miners use five postures (standing, lifting, squatting, sitting, and carrying) and all exert weight on the waist, so the probability of waist injury is unavoidable in all types of work. Long-term work and fixed night shifts also increase the incidence of low back diseases in miners [24]. Knee joint injuries may occur in coal miners who often walk long distances from their area of residence to the mining site, which increases pressure on their knee joint. A report by Weston et al. [4], on the prevalence of WMSDs in the mining industry from 2009 to 2013, showed that as working age increases, the number of leaves due to shoulder and knee pain also increases.

Results of logistic regression analysis showed that length of service, type of work, annual income, and burnout level are associated with the development of WMSDs in coal miners. This is similar to a study by Widanarko et al., which showed that coexposure of psychological and social/organizational/environmental factors increased the probability of developing WMSDs in the neck/shoulder and hand/elbow [6]. Burnout increases the development of WMSDs among coal miners. This is consistent with a study by Valadez-Torres et al. [3, 25], which showed that a high burnout level increased the risk of subsequent WMSD pain.

The average age of the miners in this study was 42.15 ± 8.65 years, but age was not associated with the prevalence of WMSDs in this study. This is different from a study by Deng et al. [17] who reported that the prevalence of WMSDs was positively correlated with age, which may be because the living quarters of miners in Xinjiang are farther from the mines than in Sichuan Province and the welfare conditions are low (the average annual income of miners in Xinjiang is 52,184 yuan), and it is difficult for coal mining companies to recruit young workers. Given that the labor force in China is aging, coal mining enterprises need to develop interventions to decrease the prevalence of WMSDs among older and senior mine workers. They also need to create awareness on preventive measures, facilitate WMSDs rehabilitation treatment, or train the miners in other skills so that they can redeploy them to other positions. A reasonable rotation system should be developed for positions with repeated operations to increase the number of rests and eliminate muscle fatigue among workers. In addition, the ground in coal mines should be improved with gravel to eliminate any unevenness or slippery sections. Furthermore, workers should be trained on effective personal protection measures. To manage moderate and severe levels of job burnout among coal miners, intervention measures such as psychological counseling should be adopted.

## 5. Conclusions

This study shows that the prevalence job burnout and WMSDs among coal miners in Xinjiang is relatively high. The shift system and gender are closely associated with job burnout. Length of service, type of work, annual income, and burnout level influence the prevalence of WMSDs. Appropriate psychological interventions should be designed to reduce job burnout, thereby the prevalence of WMSDs decreases.

## Figures and Tables

**Table 1:** The relationship between individual factors and WMSDs.

Variable	Groups	*N* (%)	WMSDs		*χ* ^*2*^	*P*	OR (95% CI)
			Sick (%)	Not sick (%)			
Gender	Male	1220 (92.1)	795 (65.2)	425 (34.8)	1.209	0.272	1
	Female	105 (7.9)	74 (70.5)	31 (29.5)			1.276 (0.826–1.973)

Ethnicity	Han Chinese	1199 (90.5)	785 (65.5)	414 (35.5)	0.072	0.788	1
	Minorities	126 (9.50)	84 (66.7)	42 (33.3)			1.055 (0.715–1.556)

Age (years)	18∼30	171 (12.9)	102 (59.6)	69 (40.4)	6.341	0.096	1
	30∼39	254 (19.2)	160 (63.0)	94 (37.0)			1.235 (0.837–1.821)
	40∼49	609 (46.0)	419 (68.8)	190 (31.2)			1.072 (0.756–1.522)
	50∼60	291 (22.0)	188 (64.6)	103 (35.4)			0.828 (0.616–1.112)

Length of service (years)	1∼10	490 (36.98)	292 (59.6)	198 (40.4)	14.493	**0.001**	1
	11∼20	277 (20.91)	182 (65.7)	95 (34.3)			1.643 (1.271–2.124)^∗∗∗^
	≥21	558 (42.11)	395 (70.8)	163 (29.2)			1.265 (0.930–1.721)

Education	Junior high school and below	581 (43.8)	371 (63.9)	210 (36.1)	1.372	0.504	1
	High school	418 (31.5)	280 (67.0)	138 (33)			1.143 (0.858–1.521)
	Bachelor and college degree and above	326 (24.6)	218 (66.9)	108 (33.1)			0.995 (0.731–1.353)

Type of work	Coal miners	258 (19.47)	183 (70.9)	75 (29.1)	11.438	**0.022**	1
	Excavate miners	316 (23.85)	185 (58.5)	131 (41.5)			0.795 (0.560–1.128)
	Transport miners	54 (4.08)	39 (72.2)	15 (27.8)			1.373 (1.001–1.885)^*∗*^
	Special miners	356 (26.87)	237 (66.6)	119 (33.4)			0.746 (0.395–1.409)
	Assist miners	341 (25.74)	225 (66.0)	116 (34.0)			0.974 (0.711–1.333)

Shift system	Day shift	490 (36.98)	330 (67.3)	160 (32.7)	6.462	**0.040**	1
	Two shifts	187 (14.11)	134 (71.7)	53 (28.3)			0.808 (0.631–1.034)
	More than two shifts	648 (48.91)	405 (62.5)	243 (37.5)			0.659 (0.873–0.941)^*∗*^

Marital status	Single	126 (9.51)	79 (62.7)	47 (37.3)	3.138	0.208	1
	Married	1066 (80.45)	694 (65.1)	372 (34.9)			1.544 (0.914–2.606)
	Divorced/separated	133 (10.04)	96 (72.2)	37 (27.8)			1.139 (0.933–2.074)

Annual income (yuan)	15000∼70000	1136 (85.74)	760 (66.9)	376 (33.1)	6.315	**0.043**	1.521 (1.090–2.124)^*∗*^
	70000∼100000	163 (12.30)	93 (57.1)	70 (42.9)			1
	100000 more than	26 (1.96)	16 (61.5)	10 (38.5)			1.263 (0.568–2.811)

*N*: number; OR: odds ratio; CI: confidence interval;^*∗*^*P* < 0.05; ^∗∗^*P* < 0.01; ^∗∗∗^*P* < 0.001. Bold P values < 0.05 are statistically significant.

**Table 2:** Comparison of job burnout grades with different demographic characteristics.

Variable	Groups	Burnout level (*n*, %)				*Z*/*Hc*	*P*
		Zero burnout	Mild burnout	Moderate burnout	Severe burnout		
Gender	Male	122 (10.0)	465 (38.1)	550 (45.1)	83 (6.8)	−3.474	**0.001**
	Female	10 (9.5)	62 (59.0)	30 (28.6)	3 (2.9)		

Ethnicity	Han Chinese	125 (10.4)	467 (38.9)	525 (43.8)	82 (6.8)	−0.520	0.603
	Minorities	7 (5.6)	60 (47.6)	55 (43.7)	4 (3.2)		

Age (years)	18∼30	19 (11.1)	65 (38.0)	82 (48.0)	5 (2.9)	1.875	0.599
	30∼39	23 (9.1)	101 (39.8)	117 (46.1)	13 (5.1)		
	40∼49	70 (11.5)	246 (40.4)	239 (39.2)	54 (8.9)		
	50∼60	20 (6.9)	115 (39.5)	142 (48.8)	14 (4.8)		

Length of service (years)	1∼10	52 (10.6)	195 (39.8)	219 (44.7)	24 (4.9)	5.821	0.054
	11∼20	38 (13.7)	108 (39.0)	116 (41.9)	15 (5.4)		
	≥21	42 (7.5)	224 (40.1)	245 (43.9)	47 (8.4)		

Education	Junior high school and below	52 (9.0)	226 (38.9)	262 (45.1)	41 (7.1)	3.789	0.150
	High school	39 (9.3)	170 (40.7)	179 (42.8)	30 (7.2)		
	Bachelor and college degree and above	41 (12.6)	131 (40.2)	139 (42.6)	15 (4.6)		

Type of work	Coal miners	23 (8.9)	85 (32.9)	132 (51.2)	18 (7.0)	6.364	0.174
	Excavate miners	36 (11.4)	123 (38.9)	137 (43.4)	20 (6.3)		
	Transport miners	5 (9.3)	21 (38.9)	27 (50.0)	1 (1.9)		
	Special miners	38 (10.7)	146 (41.0)	156 (43.8)	16 (4.5)		
	Assist miners	30 (8.8)	152 (44.6)	128 (37.5)	31 (9.1)		

Shift system	Day shift	56 (11.4)	205 (41.8)	199 (40.6)	30 (6.1)	24.095	**<0.001**
	Two shifts	9 (4.8)	52 (27.8)	111 (59.4)	15 (8.0)		
	More than two shifts	67 (10.3)	270 (41.7)	270 (41.7)	41 (6.3)		

Marital status	Single	12 (9.5)	52 (41.3)	57 (45.2)	5 (4.0)	0.515	0.773
	Married	105 (9.8)	421 (39.5)	468 (43.9)	72 (6.8)		
	Divorced/separated	15 (11.3)	54 (40.6)	55 (41.4)	9 (6.8)		

Annual income (yuan)	15000∼70000	117 (10.3)	455 (40.1)	485 (42.7)	79 (7.0)	0.680	0.712
	70000∼100000	14 (8.6)	62 (38.0)	80 (49.1)	7 (4.3)		
	100000 more than	1 (3.8)	10 (38.5)	15 (57.7)	0		

**Table 3:** Prevalence of WMSDs in different levels of burnout (*N* = 1325).

Burnout level	*N* (%)	Sick (%)	Not sick (%)	*Hc*	*P*	Adjusted OR (95% CI)
Zero burnout	132 (9.91)	89 (67.4)	43 (32.6)	17.321	**<0.001**	1
Mild burnout	527 (39.77)	349 (66.2)	178 (33.8)			2.306 (1.164–4.569)^*∗*^
Moderate burnout	580 (43.77)	359 (61.8)	221 (38.2)			2.459 (1.344–4.499)^∗∗^
Severe burnout	86 (6.49)	72 (83.7)	14 (16.3)			2.965 (1.625–5.409)^∗∗∗^
Total	1325	869 (65.58)	456 (34.42)			—

Adjustment: length of service, type of work, shift system, annual income; ^*∗*^*P* < 0.05; ^*∗∗*^*P* < 0.01; ^*∗∗∗*^*P* < 0.001.

**Table 4:** The prevalence of WMSDs in 9 locations and the distribution of different levels of job burnout.

Position	Burnout level (*n*, %)				*Hc*	*P*
	Zero burnout	Mild burnout	Moderate burnout	Severe burnout		
Neck	48 (36.4)	204 (38.7)	219 (37.7)	57 (67.1)	28.314	**<0.001**
Shoulders	48 (36.4)	151 (28.7)	195 (33.6)	54 (63.5)	40.108	**<0.001**
Back	21 (15.9)	122 (23.1)	171 (29.4)	45 (52.9)	42.833	**<0.001**
Elbows	20 (15.2)	66 (12.5)	124 (21.3)	39 (45.9)	58.023	**<0.001**
Lower back	60 (45.5)	252 (47.8)	294 (50.6)	66 (77.6)	27.880	**<0.001**
Hands/Wrists	34 (25.8)	99 (18.8)	145 (25.0)	36 (42.4)	24.193	**<0.001**
Hips	8 (6.1)	54 (10.2)	104 (17.9)	33 (38.8)	59.176	**<0.001**
Knees	42 (31.8)	160 (30.4)	193 (33.2)	53 (62.4)	34.045	**<0.001**
Ankles/Feet	23 (17.4)	92 (17.5)	137 (23.6)	37 (43.5)	31.899	**<0.001**

**Table 5:** Individual factor assignment table.

Predictor	Variable	Assignment
X1	Age	18∼29 years = 1, 30∼39 years = 2, 40∼49 years = 3, 50∼60 years = 4
X2	Length of service	1∼10 years = 1, 11∼20 years = 2, ≥21 years = 3
X3	Shift system	Day shift = 1, two shifts = 2, more than two shifts = 3
X4	Type of work	Coal miners = 1, excavate miners = 2, transport miners = 3, special miners = 4, assist miners = 5
X5	Annual income (yuan)	15000∼70000 = 1, 70000∼100000 = 2, 100000 more than = 3
X6	Burnout level	
	Zero burnout	$\begin{array}{cccc} 0 & 0 & 0 & 0 \end{array}$
	Mild burnout	$\begin{array}{cccc} 0 & 1 & 0 & 0 \end{array}$
	Moderate burnout	$\begin{array}{cccc} 0 & 0 & 1 & 0 \end{array}$
	Severe burnout	$\begin{array}{cccc} 0 & 0 & 0 & 1 \end{array}$

**Table 6:** : Multivariate logistic regression analysis of WMSDs in coal miners.

Variable	Group	Beta	Standard error	Wald	*P*	OR (95% CI)
Length of service (years)	1∼10	—	—	12.127	**0.002**	—
	11∼20	0.471	0.135	12.069	**0.001**	1.601 (1.228–2.088)
	≥21	0.204	0.161	1.614	0.204	1.226 (0.895–1.680)

Type of work	Coal miners	—	—	11.032	**0.026**	—
	Excavate miners	−0.255	0.183	1.929	0.165	0.775 (0.541–1.110)
	Transport miners	0.293	0.165	3.171	0.075	1.340 (0.971–1.850)
	Special miners	−0.373	0.329	1.284	0.257	0.689 (0.362–1.313)
	Assist miners	0.018	0.165	0.012	0.914	1.018 (0.737–1.406)

Annual income (yuan)	15000∼70000	—	—	6.642	**0.036**	—
	70000∼100000	0.442	0.174	6.434	**0.011**	1.556 (1.106–2.190)
	100000 more than	0.261	0.415	0.396	0.529	1.298 (0.576–2.929)

Burnout level	Zero burnout	—	—	14.049	**0.003**	—
	Mild burnout	0.822	0.350	5.503	**0.019**	2.275 (1.145–4.519)
	Moderate burnout	0.905	0.309	8.566	**0.003**	2.472 (1.348–4.531)
	Severe burnout	1.101	0.308	12.798	**<0.001**	3.008 (1.645–5.500)

Bold P values < 0.05 are statistically significant.

## Data Availability

All data relevant to the study are included within the article.

## Abbreviations

WMSDs: Work-related musculoskeletal disorders
OR: Odds ratio
CI: Confidence interval.

## References

[1] Occupational Safety and Health Administration (OSHA) (2001). Occupational injury and illness recording and reporting requirements, Final rule. *Federal Register*.

[2] Roquelaure Y., Bodin J., Descatha A., Petit A. (2018). Work-related musculoskeletal disorders. *La Revue du praticien*.

[3] Valadez-Torres S. G., Maldonado-Macías A. A., Garcia-Alcaraz J. L., Camacho-Alamilla M. D. R., Avelar-Sosa L., Balderrama-Armendariz C. O. (2017). Analysis of burnout syndrome, musculoskeletal complaints, and job content in middle and senior managers: case study of manufacturing industries in Ciudad Juárez, Mexico. *Work*.

[4] Weston E., Nasarwanji M. F., Pollard J. P. (2016). Identification of work-related musculoskeletal disorders in mining. *Journal of Safety, Health and Environmental Research*.

[5] Yang L., Hildebrandt V. H., Yu S. (2009). Introduction to the questionnaire for musculoskeletal disorders and the attached questionnaire. *Industrial Hygiene and Occupational Diseases*.

[6] Widanarko B., Legg S., Devereux J., Stevenson M. (2014). The combined effect of physical, psychosocial/organisational and/or environmental risk factors on the presence of work-related musculoskeletal symptoms and its consequences. *Applied Ergonomics*.

[7] Marras W. S., Cutlip R. G., Burt S. E., Waters T. R. (2009). National occupational research agenda (NORA) future directions in occupational musculoskeletal disorder health research. *Applied Ergonomics*.

[8] Eatough E. M., Way J. D., Chang C.-H. (2012). Understanding the link between psychosocial work stressors and work-related musculoskeletal complaints. *Applied Ergonomics*.

[9] Adriaenssens J., De Gucht V., Maes S. (2015). Determinants and prevalence of burnout in emergency nurses: a systematic review of 25 years of research. *International Journal of Nursing Studies*.

[10] Kim Y. J., Faber E. (2019). What medicine can teach academia about preventing burnout. *Nature*.

[11] Maslach C., Jackson S. E., Leiter M. P. (1996). *Maslach Burnout Inventory Manual*.

[12] Yong X., Gao X., Zhang Z. (2020). Associations of occupational stress with job burn-out, depression and hypertension in coal miners of Xinjiang, China: a cross-sectional study. *BMJ Open*.

[13] Davila V. J., Meltzer A. J., Hallbeck M. S., Stone W. M., Money S. R. (2019). Physical discomfort, professional satisfaction, and burnout in vascular surgeons. *Journal of Vascular Surgery*.

[14] Langballe E. M., Innstrand S. T., Hagtvet K. A., Falkum E., Aasland O. G. (2009). The relationship between burnout and musculoskeletal pain in seven Norwegian occupational groups. *Work*.

[15] Liu F.-D., Pan Z.-Q., Liu S.-L. (2017). The estimation of the number of underground coal miners and normalization collective dose at present in China. *Radiation Protection Dosimetry*.

[16] Lu Y., Zhang Z., Gao S., Yan H., Zhang L., Liu J. (2020). The status of occupational burnout and its influence on the psychological health of factory workers and miners in Wulumuqi, China. *BioMed Research International*.

[17] Deng M., Wu F., Wang J., Sun L. (2017). Musculoskeletal disorders, personality traits, psychological distress, and accident proneness of Chinese coal miners. *Work*.

[18] Li F., Liu J., Lian Y. (2009). Reliability and validity of job burnout measurement tool in knowledge worker. *Chinese Journal of Occupational Diseases of Labour Hygiene*.

[19] Li F. (2008). *A Study on the Relationship between Occupational Stress and Job Burnout and its Influencing Factors in Knowledge Worker*.

[20] Du W., Wang S., Wang J. (2012). Evaluation of reliability and validity of the questionnaire on musculoskeletal disorders. *Chinese Journal of Occupational Diseases in Occupational Health*.

[21] Xu G.-X., Li L.-P., Liu F.-Y., Wang S. (2012). Relationships between psychosocial factors and work-related musculoskeletal disorders in coal miners. *Chinese Journal of Industrial Hygiene and Occupational Diseases*.

[22] Yan P., Li F., Zhang L. (2017). Prevalence of work-related musculoskeletal disorders in the nurses working in hospitals of Xinjiang Uygur autonomous region. *Pain Research & Management*.

[23] Bauernhofer K., Bassa D., Canazei M. (2018). Subtypes in clinical burnout patients enrolled in an employee rehabilitation program: differences in burnout profiles, depression, and recovery/resources-stress balance. *BMC Psychiatry*.

[24] Widanarko B., Legg S., Devereux J., Stevenson M. (2015). Interaction between physical and psychosocial work risk factors for low back symptoms and its consequences amongst Indonesian coal mining workers. *Applied Ergonomics*.

[25] Armon G., Melamed S., Shirom A., Shapira I. (2010). Elevated burnout predicts the onset of musculoskeletal pain among apparently healthy employees. *Journal of Occupational Health Psychology*.

